# Moral Injury and Suicide Ideation Among Combat Veterans: The Role of Trauma-Related Shame and Collective Hatred

**DOI:** 10.1192/j.eurpsy.2022.2187

**Published:** 2022-09-01

**Authors:** Y. Levi-Belz, G. Zerach, G. Schwartz, E. Halperin

**Affiliations:** 1 Ruppin Academic Center, The Lior Tsfaty Center For Suicide And Mental Pain Studies, Emek Hefer, Israel; 2 Ariel University, Psychology, Ariel, Israel

**Keywords:** trauma-related shame, Moral Injury, suicide ideation, Veterans

## Abstract

**Introduction:**

Exposure to potentially morally injurious events (PMIEs) among combat veterans has been acknowledged as a significant stressful combat event that may lead to mental health problems, including suicide ideation (SI). Several studies have examined the risk and protective factors that can explain the conditions in which PMIEs may contribute to the development and maintenance of SI. However, the contribution of social-emotional factors has yet to be examined.

**Objectives:**

In the current study, we examined the association between PMIE-Self and SI among combat veterans and explored the mediating role of trauma-related shame and the moderation role of collective hatred in this association.

**Methods:**

A volunteer sample of 336 Israeli combat veterans was recruited, completing self-report questionnaires in a cross-sectional study.

**Results:**

indicated that PMIE-Self was positively associated with SI, and trauma-related shame mediated this association. Moreover, collective hatred moderated both their direct (PMIE -SI) and indirect (PMIE-Shame-SI) association. Notably, collective hatred had an inverse role for each of the associations. Thus, collective hatred was found to comprise both a risk and a protective factor for SI following PMIE-Self.

**Conclusions:**

The current findings highlight the crucial contribution of trauma-related shame and collective hatred to the association between moral injury and suicidality. Moreover, the findings demonstrate that even years after their military service release, combat veterans exposed to PMIEs may still feel consumed by painful memories and maintain premonitions of a foreshortened future. Furthermore, the findings help to better understand the dynamics of collective hatred and the challenge of modifying it.

**Disclosure:**

No significant relationships.

